# Understanding the relationship between HCV infection and progression of kidney disease

**DOI:** 10.3389/fmicb.2024.1418301

**Published:** 2024-06-28

**Authors:** Meiqi Zhang, Zhongyu Han, Yumeng Lin, Zi Jin, Shuwei Zhou, Siyu Wang, Yuping Tang, Jiaxuan Li, Xueping Li, Haoran Chen

**Affiliations:** ^1^School of Medical and Life Sciences, Chengdu University of Traditional Chinese Medicine, Chengdu, China; ^2^Naniing Tongren Hospital, School of Medicine, Southeast University, Nanjing, China; ^3^Department of Anesthesiology and Pain Rehabilitation, Shanghai YangZhi Rehabilitation Hospital (Shanghai Sunshine Rehabilitation Center), School of Medicine, Tongji University, Shanghai, China; ^4^Jiangsu Key Laboratory of Molecular and Functional Imaging, Department of Radiology, Zhongda Hospital, School of Medicine, Southeast University, Nanjing, China; ^5^Department of Gastroenterology, The First Hospital of Hunan University of Chinese Medicine, Changsha, China; ^6^Hepatobiliary Department of the Third Affiliated Hospital of Guangxi University of Traditional Chinese Medicine, Nanning, Guangxi, China; ^7^School of Basic Medical Sciences, Fujian Medical University, Fuzhou, China; ^8^School of Basic Medical Sciences, Chengdu University of Traditional Chinese Medicine, Chengdu, China; ^9^Department of General Surgery, Chengdu Xinhua Hospital Affiliated to North Sichuan Medical College, Chengdu, China

**Keywords:** hepatitis C virus, cryoglobulinaemia, acute kidney injury, glomerulonephritis, diabetic nephropathy, lupus nephritis, renal cell carcinoma

## Abstract

Hepatitis C virus (HCV) can cause a range of kidney diseases. HCV is the primary cause of mixed cryoglobulinaemia, which leads to cryoglobulinaemic vasculitis and cryoglobulinaemic glomerulonephritis (GN). Patients with acute cryoglobulinaemic vasculitis often exhibit acute kidney disease due to HCV infection, which typically progresses to acute kidney injury (AKI). HCV also increases the risk of chronic kidney disease (CKD) and the likelihood of developing end-stage renal disease (ESRD). Currently, direct-acting antiviral agents (DAAs) can be used to treat kidney disease at different stages. This review focuses on key findings regarding HCV and kidney disease, discusses the impact of DAAs, and highlights the need for further research and treatment.

## Methods

We conducted a systematic literature search using PubMed, EMBASE, Web of Science, and CENTRAL within the Cochrane Library without date or language limitations. The search terms used were “(Hepatitis C virus OR HCV) AND (cryoglobulinaemia OR CG),” “(Hepatitis C virus OR HCV) AND (acute kidney injury OR AKI),” “(Hepatitis C virus OR HCV) AND (glomerulonephritis OR GN),” “(Hepatitis C virus OR HCV) AND (diabetic nephropathy OR DN OR DKD),” “(Hepatitis C virus OR HCV) AND (lupus nephritis OR LN),” “(Hepatitis C virus OR HCV) AND (renal cell carcinoma OR RCC).”

## Introduction

More than 71 million people worldwide have chronic HCV, which is endemic in all regions; moreover, the incidence of HCV continues to increase each year (Berggren et al., 2020). HCV primarily replicates in liver cells, and identification of virus-infected liver cells by the immune system leads to an increase in necrotizing inflammation and fibrosis, which ultimately results in cirrhosis (Abu-Freha et al., 2022).

**Figure 1:**
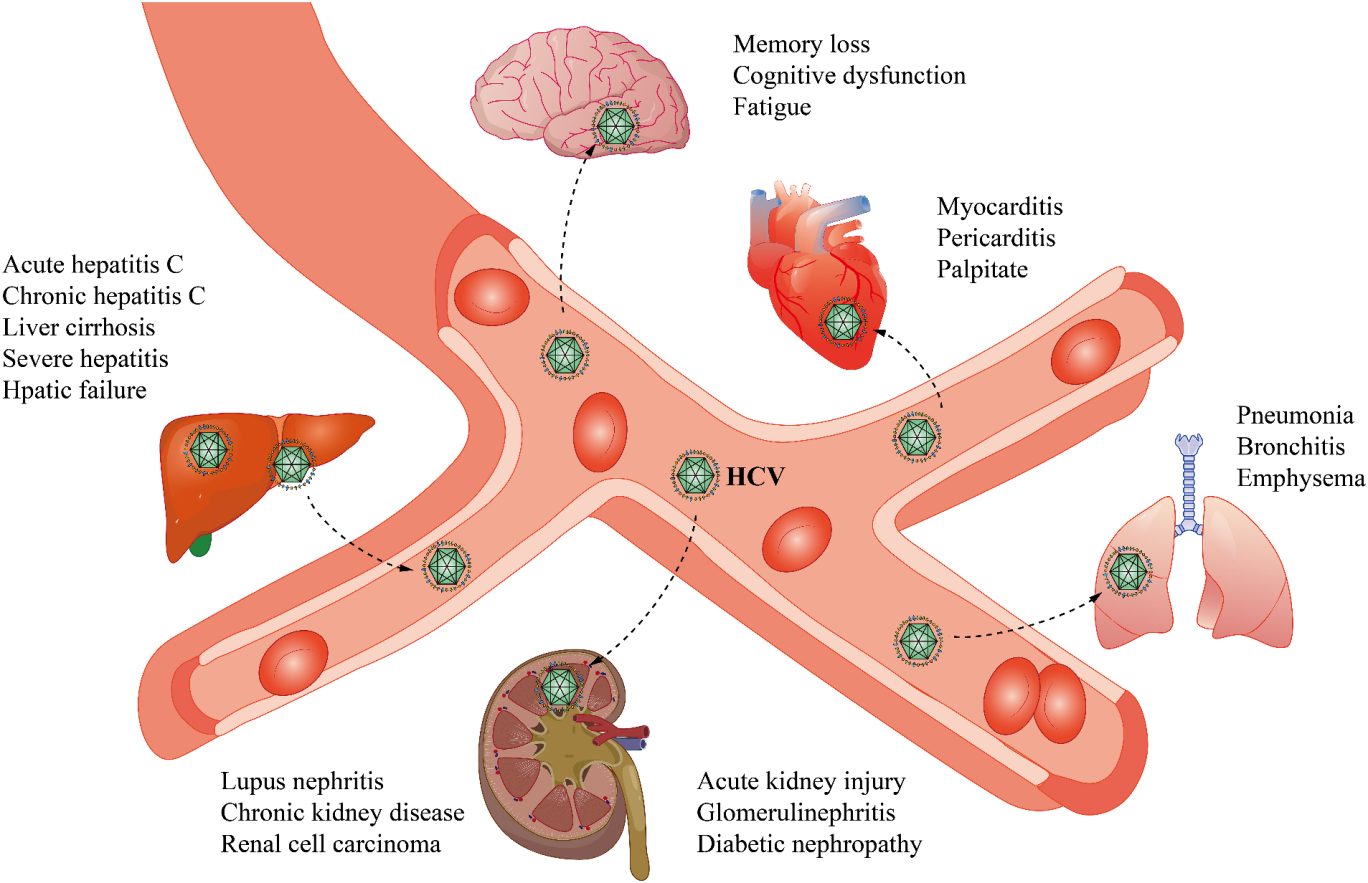
HCV infection not only affects the liver but also has many extrahepatic manifestations. In the liver, the effects of HCV include acute and chronic hepatitis C, cirrhosis, severe hepatitis, and hepatic failure. In the brain, memory loss, cognitive dysfunction, changes in brain metabolism, and fatigue may occur. In the heart, cardiovascular disease, myocarditis, pericarditis, and palpitations may be caused by HCV. HCV may also cause some lung diseases, such as pneumonia, bronchitis, and emphysema. In the kidney, HCV causes acute kidney injury, glomerular nephropathy, diabetic nephropathy, lupus nephritis, chronic kidney disease, and renal cell carcinoma.

HCV can damage multiple organs throughout the body and can cause diseases such as kidney disease (Figure 1). Excessive cryoglobulin production during HCV infection may trigger immune complex-mediated vasculitis and induce inflammation caused by vascular thrombosis and cryoglobulin deposition, which can progress to acute kidney injury (AKI; Satapathy et al., 2014). HCV is closely related to membranoproliferative glomerulonephritis (MPGN), which is often accompanied by type II mixed cryoglobulinaemia (MC; Dammacco et al., 2007). With respect to diabetic nephropathy, HCV infection rates are highest in patients with type II diabetes-related glomerulosclerosis (II-DGS; Soma et al., 2000). HCV infection is linked to the occurrence of autoimmune diseases, such as lupus nephritis (LN), in the kidney (Kirou et al., 2022). Several studies in recent years have reported that individuals with HCV have a higher risk of developing chronic kidney disease (CKD) than uninfected individuals (Barsoum et al., 2017). In addition, individuals who are infected with HCV have a significantly greater risk of developing renal cell carcinoma (RCC; Wijarnpreecha et al., 2016). Significant advancements in drug development have allowed HCV infections to be treated effectively with combinations of direct-acting antiviral agents (DAAs). In this manuscript, we determine the connection between HCV and various kidney diseases, elaborate on the possible mechanisms of some kidney diseases and suggest therapeutic drugs and treatments.

## Hepatitis C virus

**Figure 2:**
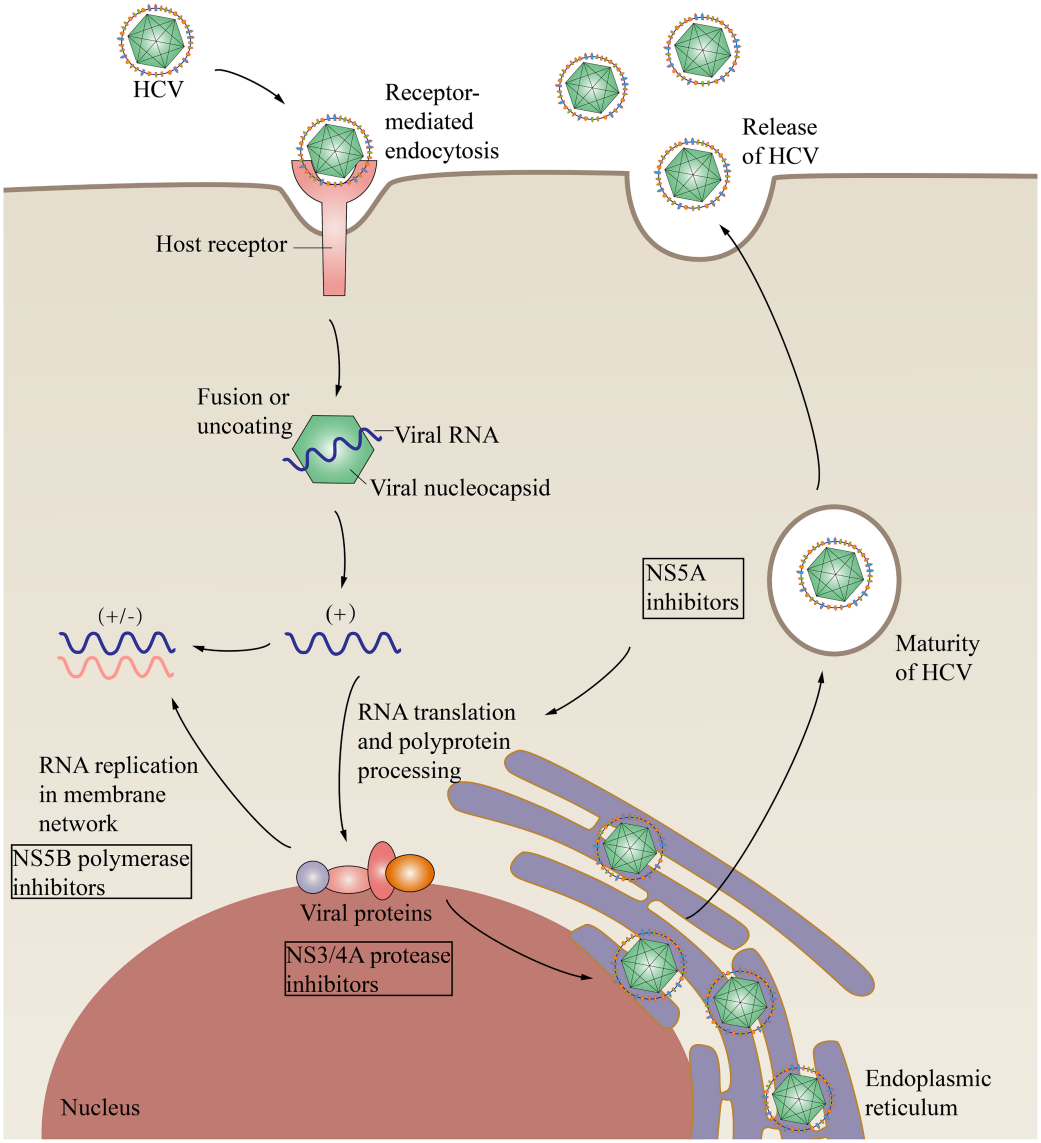
HCV replication within cells and the effects of direct-acting antivirals. HCV binds to cell surface receptors and undergoes endocytosis. Membrane fusion results in the release of the nucleocapsid containing viral RNA into the cytoplasm. RNA is replicated in the membrane network and is then translated, forming a large polymeric protein. NS3/NS4 proteases include NS3/4A proteases, NS5B polymerases that function in HCV RNA replication, and multifunctional NS5A, which is involved in HCV replication and cleaves polymers into structural components and nonstructural proteins. Direct-acting antivirals (DAAs) can inhibit HCV replication and processing. NS3/4A, Nonstructural protein 3/4A; NS5A, Nonstructural protein 5A; NS5B, Nonstructural protein 5B.

HCV virions are spherical, capsular, approximately 55–65 nm in diameter, and contain single-stranded, positive-sense RNA approximately 9.5 kb in length. The genome consists of a 5′ noncoding region, a coding region, and a 3′ noncoding region (Adams et al., 2017). Immediately downstream of the 5′ noncoding region is an open reading frame (ORF), in which the genome is arranged in the order 5′-C-E1-E2/NS1-NS2-NS3-NS4-NS5-3′. The glycoproteins E1 and E2/NS1 can neutralize HCV (Tsukiyama-Kohara and Kohara, 2017). The functions of NS2 and NS4 are vague, but both of these proteins are tightly bound to the cell membrane. The helicase activity of NS3 assists in RNA replication, and NS5, with its RNA-dependent polymerase activity, is involved in the replication of the HCV genome (Tabata et al., 2020; Figure 2). The function of the noncoding region at the 3′ end is unclear and may be related to viral replication.

The transmission route of HCV is primarily through blood transfusion or transfer of blood products. In addition, hidden minor trauma, sexual contact, close household contact, and vertical transmission can be achieved through nontransfusion routes (Dearborn and Marcotrigiano, 2020).

## HCV in renal diseases

### Renal microenvironment

The kidney plays a particularly significant role in metabolic activities and is an important organ for maintaining the internal environment and excreting metabolic waste (Fazelian et al., 2021). The renal microenvironment includes many types of cells, including immune cells and intrinsic renal cells, which together, constitute this complex system (Han et al., 2022). Immune cells include macrophages, dendritic cells (DCs), T cells and B cells. Intrinsic renal cells include renal tubular epithelial cells (RTES), podocytes, endothelial cells (ECs), renal pericytes, and mesangial cells (MCs; Han et al., 2022).

**Figure 3:**
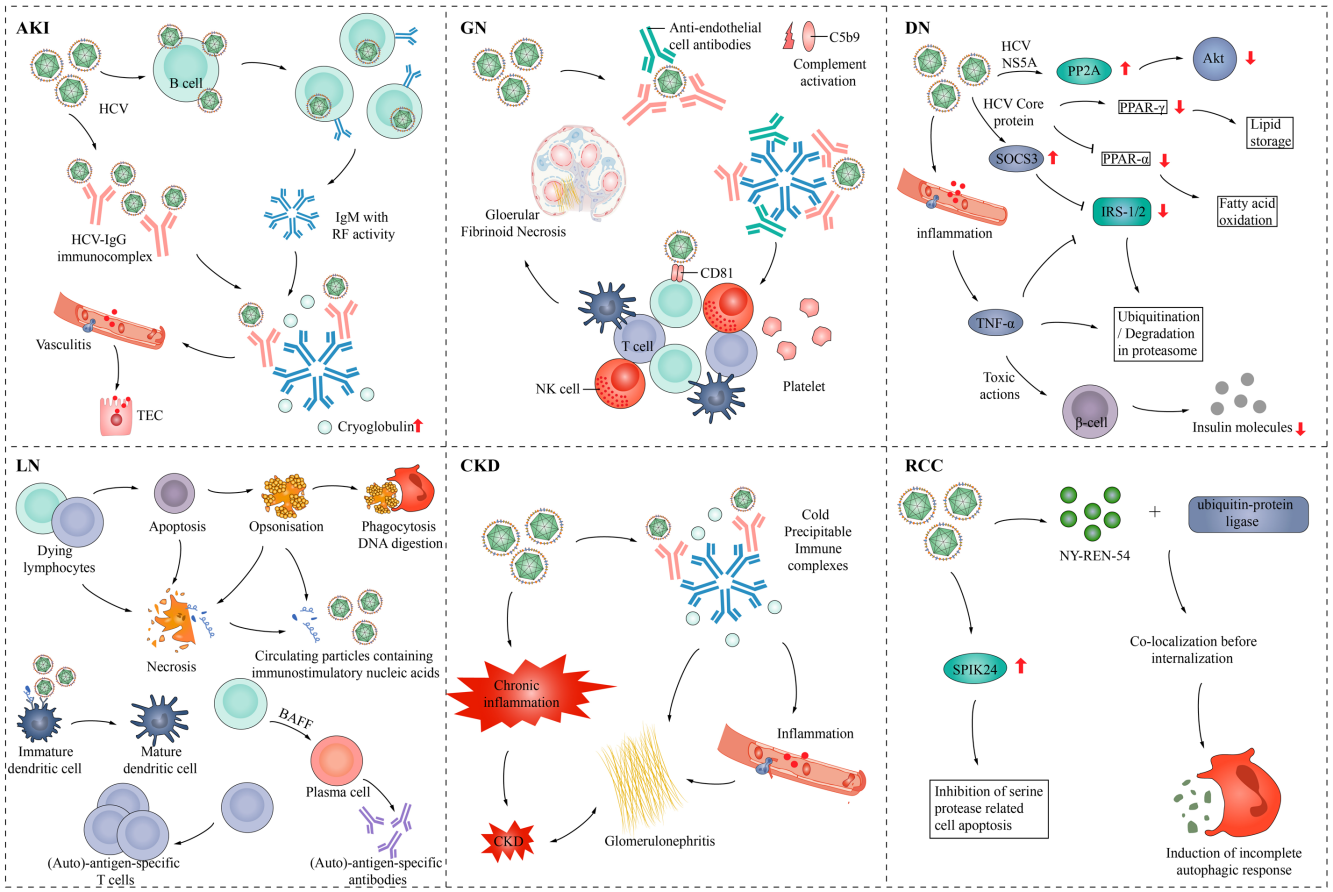
Pathogenesis of HCV infection in various kidney diseases. AKI, Acute kidney injury; Akt, Protein kinase B; BAFF, B-cell activating factor; CKD, Chronic kidney disease; DN, Diabetic nephropathy; GN, Glomerulonephritis; IgG, Immunoglobulin G; IgM, Immunoglobulin M; NK cell, Natural killer cell; NS5A, Nonstructural protein 5A; PPAR-α, peroxisome proliferator-activated receptor α; PPAR-γ, Peroxisome proliferator-activated receptor γ; PP2A, Protein phosphatase 2; RCC, Renal cell carcinoma; RF, rheumatoid factor; SPIK24, Serine protease inhibitor Kazal protein 24; SOCS3, Suppressor of cytokine signaling 3; TEC, Tubular epithelial cell; TNF-α, Tumor necrosis factor-α; IRS-1/2, Insulin receptor substrate 1/2.

HCV causes various types of damage to the kidney (Figure 3). HCV infection can have adverse effects on the kidneys, primarily through the following two mechanisms: damage to the kidney tissue caused by the immune system, typically cryoglobulinaemia, and direct impact of the virus on the kidneys (Pol et al., 2019).

Three types of cryoglobulinaemia have been described. Type I cryoglobulinaemia is always associated with B-cell proliferative disorders and is characterized by high levels of monoclonal immunoglobulin M (IgM) and rarely immunoglobulin G (IgG) or immunoglobulin A (IgA) cryoglobulins. Types II and III are mixed types of cryoglobulinaemia that are associated with HCV infection, autoimmune diseases or B-cell proliferative disorders (Roccatello et al., 2018). The most important cause is chronic HCV infection, which accounts for 80%–90% of all cases (Leslie et al., 2022).

CD81 is a receptor for HCV that is expressed on liver cells and B lymphocytes and possibly by cells within kidney tissue (Yamamoto et al., 2020). HCV binds to multiprotein complexes consisting of CD81, CD21 and CD19 on the surface of B cells and can lower the threshold for B-cell activation, which leads to the production of many types of autoantibodies. Oligoclonal monotypic lymphocyte proliferation in mixed cryoglobulinaemia is maintained by HCV-dependent gene translocation, which protects cells from apoptosis.

HCV can also induce lysosomal enzyme abnormalities in macrophages, rendering immune complexes indigestible after phagocytosis (Costa-Verdera et al., 2021). In the kidney, the accumulation of immune complexes triggers glomerular inflammation, which activates anti-HCV IgG antibodies, anti-endothelial antibodies and the complement system.

In addition, HCV can damage tissues through direct infection of endothelial cells, tubular epithelial cells, and renal-infiltrating white blood cells (Biliotti et al., 2021; Grossini et al., 2023). Endothelial cells that are infected can undergo apoptosis, similar to HCV-infected endothelial cells within the blood–brain barrier (Fletcher et al., 2012). HCV can also infect mesangial cells, leading to chemokine activation (Zito et al., 2022). In addition, individuals with HCV infection may face increased risks of cardiovascular, cerebral, or renovascular events as well as a greater likelihood of developing insulin resistance and type 2 diabetes than those not infected with HCV (Fukui et al., 2003; Mostafa et al., 2010; Adinolfi et al., 2012; Petta et al., 2014; Negro et al., 2015; Hwang et al., 2016).

## HCV in AKI

AKI is characterized by a rapid deterioration in kidney function within a brief period. This decline is attributed to a sudden or persistent decrease in the glomerular filtration rate (Pickkers et al., 2021). AKI is characterized by an increase in the serum creatinine level of 0.3 mg/dL or a reduction in urine production of 0.5 mL/kg/h (Kellum et al., 2021). Due to its high initial mortality rate, morbidity, and substantial health care costs, AKI remains a significant health burden (Kellum et al., 2021). According to a database of 500,000 patients in western Pennsylvania, the incidence of AKI was found to be 12%. The annual incidence rate of AKI in the US is estimated to be approximately 4.2 million cases, while approximately 35 million Americans are hospitalized each year due to AKI. This number can be extrapolated to nearly 98 million cases worldwide (Peerapornratana et al., 2019).

In patients with acute cryoglobulinaemic vasculitis, HCV can trigger AKI (Barsoum et al., 2017). Cryoglobulinaemic vasculitis is a systemic disease involving multiple organs that, primarily affects the skin, joints, peripheral nervous system and kidneys (Sethi et al., 2022). HCV is the primary cause of mixed cryoglobulinaemic vasculitis. In the kidney, cryoglobulinaemic vasculitis is characterized by subendothelial deposition, which is visible under immunofluorescence, diffuse IgM deposition in capillary pores, and formation of intimal thrombi composed of cryoglobulin deposits. The primary mechanism of vascular injury is the complement protein complex C1q, and anti-HCV IgG antibodies stimulate an IgM-rheumatoid factor antibody response, which immobilizes and activates complement through the classical pathway (Boleto et al., 2021). Binding of C1q to receptors on endothelial cells localizes immune complexes to the renal capillary bed. Through the complement cascade, C4b C2b (C3 convertase) is formed, and C3 is cleaved into C3a and C3b. C3b acts as a C5 convertase that induces cleavage of C5 into C5a and C5b. C3a and C5a are chemotactic and recruit neutrophils that trigger an inflammatory response. This cascade also leads to the formation of C5-9, a membrane attack complex that may be important in endothelial injury (Barsoum et al., 2017).

One study examined 468 people with chronic HCV infection, 63 patients experienced a total of 124 AKI events from 3 months to 6 months. Patients with AKI exhibited a markedly greater occurrence of comorbidities such as diabetes mellitus (47.6% vs. 16.5%, *p* < 0.0001) and hypertension (69.8% vs. 38.8%, *p* = 0.0005). Among the diabetic patients, 31% (30 out of 97) experienced AKD events, whereas only 8.9% (33 out of 371) of nondiabetic patients experienced the same events (*p* < 0.0001). Based on a 13-year follow-up study, a total of 1,252 patients diagnosed with HCV infection were included in a retrospective study conducted at two medical centers. Among these patients, 1,008 were categorized as having chronic hepatitis (CH), 123 with compensated cirrhosis (Com-LC), and 121 with decompensated cirrhosis (decomp-LC) or hepatocellular carcinoma (HCC). Over an average follow-up period of 5.2 years, 285 patients experienced AKI with an incidence rate of 4.35 per 100 person-years. The occurrence of AKI was observed to rise progressively with the advancement of chronic hepatitis C: CH (3.32/100 person-years), Com-LC (5.86/100 person-years), and decomp-LC or HCC (17.28/100 person-years). The 14-year survival rate was notably higher for patients without AKI compared to those with AKI (94.2% vs. 26.3%, *p* < 0.001). The presence of AKI was identified as common among individuals with chronic HCV infection and was linked to a substantial increase in overall mortality (Jeon et al., 2021). In addition, most AKI patients were also HCV-positive. Nausea, vomiting, diarrhea, dehydration, and large volume paracentesis are the main causes of AKI attacks (Satapathy et al., 2014). Mass paracentesis involves the removal of >4–5 L of fluid, which may induce AKI by worsening vasodilation and intravascular depletion (Cullaro et al., 2022). Hepatic encephalopathy may also indirectly affect the development of AKI (Satapathy et al., 2014).

Cirrhosis will develop in an estimated 5%–20% of patients infected with HCV, and hepatocellular carcinoma will develop in approximately 1%–4% of patients (Ali et al., 2023). AKI often occurs in individuals diagnosed with cirrhosis, primarily because of the typical hemodynamic abnormalities observed in patients with cirrhosis and ascites (Kirita et al., 2020). Elevated portal venous pressure results in the development of alternative pathways for blood flow within the body, as well as a simultaneous expansion of blood volume in both the internal organs and the entire circulatory system. This expansion causes a decrease in the effective volume of blood flowing through the arteries, which subsequently triggers responses in the neurosensory system, particularly the renin-angiotensin-aldosterone system (RAAS) and the sympathetic nervous system, and induces the release of antidiuretic hormone without osmotic concerns. The retention of sodium, increase in blood volume, heightened circulation characterized by low resistance in systemic blood vessels, and the increase in the volume of blood pumped by the heart are the outcomes of the activation of the RAAS and the sympathetic nervous system (Schrier et al., 1988; Belcher et al., 2013a). The primary reason for vasodilation in patients with cirrhosis is the increased production of nitric oxide (NO; Hill et al., 2021). Most occurrences of acute kidney injury in cirrhosis patients occur outside the hospital. These patients often experience fluctuations in their blood volume caused by the use of diuretics and diarrhea caused by lactulose (de Carvalho et al., 2012; Belcher et al., 2013a,b). In cirrhosis, only approximately one-third of AKI cases are caused by intrinsic kidney disease, specifically acute tubular necrosis (ATN). This differs from hypoperfusion, which is the leading cause of AKI in cirrhosis patients, as it accounts for 68% of cases. Among patients with hypoperfusion-associated AKI, approximately 45% of these AKI cases exhibit improvement with increased volume and are classified as prerenal azotaemia (PRA). The remaining 1/3, or 23% of all AKI cases, do not respond to the increased volume, and these patients are diagnosed with hepatorenal syndrome (HRS). Systemic disorders such as glomerulonephritis may also occur (Belcher et al., 2013b).

## HCV in GN

Glomerulonephritis (GN) encompasses a range of kidney diseases that damage the glomeruli through immune-mediated damage. Complement system disruptions largely contribute to the various manifestations of these diseases. These disruptions can arise from genetic defects, autoimmunity, microorganisms, or abnormal immunoglobulins, such as altered IgA or paraproteins. The common thread among these problems is the excessive or misguided activation of alternative pathways in the complement system (Kaartinen et al., 2019). Different histological patterns of natural renal GN have been reported to be linked to HCV infection. The most prevalent type of HCV-associated GN is MPGN, which is associated with type II cryoglobulinaemia (Fenoglio et al., 2022). MPGN without cryoglobulinaemia, membranous glomerulonephritis, focal segmental glomerulosclerosis, endocapillary proliferative glomerulonephritis, renal thrombotic microangiopathy related to anticardiolipin antibodies, fibrillary glomerulopathy and immunotactoid glomerulopathy are less common (Johnson et al., 1994; Stehman-Breen et al., 1999).

MPGN is a form of glomerulonephritis that is caused by immune complex deposition (Noris et al., 2023). Depending on the cause, MPGN can be categorized as primary or secondary, with secondary MPGN often occurring as a result of infection with hepatitis B or C virus, which causes persistent antigenaemia and the formation of antigen–antibody immune complexes that are deposited in the glomeruli (Kanduri et al., 2024). A previous study revealed that between 2001 and 2006, 126 patients were diagnosed with MPGN at the Mayo Clinic. Twenty patients who had not been assessed for HBV or HCV infection were excluded. Among the remaining 106 patients, approximately 23.5% (25 patients) tested positive for either hepatitis B or C or both. Specifically, 12 patients were positive for hepatitis B, 13 patients were positive for hepatitis C, and 2 patients tested positive for both hepatitis B and C (Sethi et al., 2010). In addition, the prevalence of HCV seropositivity in the MPGN case series in the United States is approximately 10-fold greater than the national HCV prevalence (Johnson et al., 1993; Rennke, 1995; Yamabe et al., 1995). In a series of biopsies and autopsies, MPGN was found to be more common in HCV-infected individuals than in the general population (Gopalani and Ahuja, 2001; el-Serag et al., 2002).

Type II MC leading to MPGN is associated with HCV infection (Dammacco et al., 2007). HCV replicates in B lymphocytes, which can trigger cryoglobulinaemia (Moretti et al., 2024). In at least 4/5 of cases, patients diagnosed with mixed cryoglobulinaemia exhibit HCV infection, and all patients with HCV-associated MPGN exhibit symptoms of cryoglobulinaemia (Retamozo et al., 2022). In related experimental studies, the underlying cause of type II cryoglobulin-associated nephritis has been suggested to be selective binding between the Ig kappa rheumatoid factor (RF) component and the mesangial matrix. The primary method used to determine this potential binding is to study the binding capacity of type II cryoglobulin IgM kappa RFs to various types of monoclonal and polyclonal IgM and IgM RFs with distinct characteristics and structures. Thirteen IgMs were isolated from human IgM kappa/IgG cryoglobulin, eight monoclonal IgMs were isolated from macroglobulinemia patients, nine polyclonal IgMs were isolated from healthy donors, and eight polyclonal IgM RFs were isolated from rheumatoid arthritis patients. These purified IgM samples were subjected to enzyme-linked immunosorbent assay (ELISA) on cFN-coated plates at the same concentration. All cryoglobulin IgMs exhibited strong binding to cFN, whereas IgMs from macroglobulinemia, normal IgMs, and polyclonal IgM RFs displayed either weak or no binding. The binding of IgM kappa to cFN remained intact when IgM kappa monomers were used, and in the inhibition test, specific inhibition was observed only with cFN and not with plasma FN. RFs exhibited dose-dependent binding to IgG. These findings indicate that the strong attraction between cryoglobulin IgM kappa RF and immobilized cFN could contribute to the high nephrogenicity of type II cryoglobulins and may contribute to the formation of immune complexes *in situ* (Fornasieri et al., 1996). In addition, the association of HCV infection with hematological malignancies, mainly non-Hodgkin’s lymphoma, has been supported by several studies. For these reasons, the association between known systemic autoimmune diseases and HCV infection, coupled with the potential for any of these diseases to develop into B-cell non-Hodgkin’s lymphoma, suggests that there may also be a close relationship between Sjogren syndrome (SS), HCV, and B-cell lymphoproliferative disorders. This is especially the case in patients with type II mixed cryoglobulinemia (Treppo et al., 2021).

Furthermore, studies indicated that individuals with glomerulonephritis who are infected with HCV have a greater likelihood of CKD progression. Moreover, this risk is even more significant for patients who also have diabetic nephropathy (Noureddine et al., 2010).

## HCV in diabetic nephropathy

Glomerular pathology in diabetic nephropathy can be categorized into four different grades: class I, thickening of the glomerular basement membrane; class II, mild (IIa) or severe (IIb) mesangial dilatation; class III (tuberous sclerosis or Kimmelstiel-Wilson disease), in which at least one glomerulus exhibits increased mesangial stromal nodularity (Kimmelstiel-Wilson); and class IV (advanced diabetic glomerulosclerosis), >50% total glomerulosclerosis (Tervaert et al., 2010).

HCV infection has been reported in previous studies to have a significantly high prevalence in patients with type II diabetic-related nephropathy, which may adversely affect disease progression (Soma et al., 2000; Naing et al., 2013; Cullaro et al., 2022). In a study of 2,370 individuals who underwent biopsies and who were able to undergo HCV serology testing, the highest rates of HCV infection were observed in patients with II-DGS. Among 123 patients with II-DGS, 24 (19.5%) were infected with HCV. Additionally, HCV infection was assessed in a random sample of 545 individuals with type II diabetes, including both outpatients and inpatients, who did not undergo a kidney biopsy. Among this group, 56 patients (10.3%) were found to be positive for HCV antibodies, and the level of proteinuria in these HCV-positive patients was greater than that in the 489 patients who tested negative for HCV (Soma et al., 2000). In a comprehensive study involving 642 participants (498 who tested positive for anti-HCV and 144 who tested negative), HCV-infected patients exhibited a greater prevalence of impaired fasting blood glucose (IFG), irrespective of their diabetic status (Lecube et al., 2006). According to one study, the prevalence of HCV was 2% in individuals with type II diabetes and 0.33% in nondiabetic individuals group (Makvandi et al., 2021). Research has also indicated that individuals with HCV who have immune-inflammatory II-DGS experience a more rapid decline in renal function than II-DGS patients without HCV infection (Soma et al., 2000). Other reports have indicated a correlation between HCV infection and the development of posttransplant diabetes mellitus (PTDM) following liver and kidney transplantation. PTDM is a frequently occurring problem following organ transplantation that significantly impacts the long-term survival of both the recipient and the transplanted organ (Knobler et al., 1998; Baid et al., 2001, 2002; Kasiske et al., 2003; Kellum et al., 2021). In a cross-sectional study, 65 cases of patients with kidney damage were compared based on clinical and histopathological features. The main study group consisted of 20 diabetic patients with hepatitis C virus (DM-HCV), 20 patients with HCV and 25 diabetic patients without HCV were included as the disease control group. The prevalence of glomerulosclerosis was notably higher in patients with both diabetes and hepatitis C (with a median of 44.5%) compared to those in the diabetes group (7%) and hepatitis C group (7%). The DM-HCV group exhibited a tendency toward diffuse (20%) and global (75%) sclerosis, along with moderate-to-severe tubular atrophy (45%) and interstitial fibrosis (55%). Electron microscopy revealed a significantly higher occurrence of podocyte lesions in the DM-HCV group (70%) vs. the DM group (12%; Samaan et al., 2022).

Both the degree of proteinuria and severe arteriolar hyalinosis have been linked to decreased kidney function in patients with II-DGS. Research has demonstrated that compared with HCV-negative individuals, HCV-positive individuals exhibit elevated proteinuria. Additionally, in one study, the serum creatinine level in HCV-positive patients was 0.97 ± 0.76 mg/dL, whereas in HCV-negative patients, the serum creatinine level was 0.85 ± 0.71 mg/dL (Soma et al., 2000), which suggests that HCV infection may influence the progression of diabetic nephropathy. Immune complex glomerulonephritis occurs more frequently in patients with diabetic nephropathy than in nondiabetic individuals (Appel et al., 1983; O'Neill et al., 1983; Cavallo et al., 1984). According to one study, patients who tested positive for HCV had a greater likelihood of also having grade II DGS than patients who tested negative for HCV. Among 99 patients, 9.1% (9 patients) who tested positive for HCV had grade II DGS in addition to 3 patients with MPGN and 1 patient with MN, whereas the HCV-negative group included 6 patients with IgAN and no patients with MPGN (Simó et al., 1996). Based on the observed correlation between HCV infection and MPGN, it is believed that the association between MPGN and MN in patients with II-DGS may be linked to HCV infection. Additionally, some studies have proposed that HCV can infect islet cells, which can directly damage B cells (Simó et al., 1996). Patients with HCV infection exhibit increased levels of fasting serum insulin and insulin resistance, as well as a greater incidence of diabetes (Ciardullo et al., 2022). This is because HCV core proteins play a direct role in reducing the expression of insulin receptor substrate (IRS) proteins 1 and 2 (Ciardullo et al., 2022).

The number of patients who require hemodialysis due to diabetic nephropathy has significant increased (Selby and Taal, 2020). Causes include poor blood sugar control, increased blood pressure, proteinuria, lipid abnormalities and genetic factors (Selby and Taal, 2020). The Centers for Disease Control and Prevention (CDC) issued a health advisory highlighting a rise in cases of acute HCV infection in patients undergoing ongoing hemodialysis treatment. A positive serologic status for anti-HCV is strongly linked to decreased survival rates in individuals undergoing dialysis (Cacoub et al., 2016). We speculate that HCV can be transmitted through equipment used in dialysis (Martin and Fabrizi, 2008; Cacoub et al., 2016). The cause of the high incidence of HCV infection in individuals with diabetic nephropathy is currently unknown, but it may be related to spread via dialysis equipment.

The Dialysis Outcomes and Practice Patterns Study (DOPPS) is a prospective observational study conducted in 2004 that involved randomly selected adult hemodialysis patients from 308 dialysis facilities in Europe and the United States (Fissell et al., 2004). This study revealed that the average prevalence of HCV infection in these facilities was 13.5% and that the prevalence ranged from 2.6% to 22.9% across different countries. Factors such as longer dialysis duration, male sex, black race, diabetes, HBV infection, prior kidney transplantation, and substance abuse were associated with a greater incidence of HCV infection. Seroconversion rates were linked to an increase in HCV incidence during treatment (RR = 1.36, *p* < 0.0001) but not to the isolation of HCV-infected patients (RR = 1.01, *p* = 0.99; Fissell et al., 2004). Another DOPPS study involving 49,762 hemodialysis patients from 1996 to 2011 reported a 9.5% HCV seropositivity rate (Goodkin et al., 2013). In a national prospective cohort from France with 72,948 patients initiating dialysis or undergoing preemptive kidney transplantation, a low HCV infection rate of 0.84% (95% CI, 0.78 to 0.91) was observed. The frequency of HCV infection among patients who undergo hemodialysis varies but remains consistently higher than that in the general population.

A study conducted in Portugal reported that reusing dialysers increases the risk of HCV seroconversion. Dialysis centers that reused dialysers and had a designated room for reprocessing dialysers used in patients with anti-HCV antibodies exhibited notably lower seroconversion rates than centers that did not have these specific precautions in place (Pinto dos Santos et al., 1996). The reason behind this discrepancy, whether it indicates a causal connection or simply better compliance with infection control measures in centers that segregate dialyser reuse practices for HCV-positive patients, remains uncertain.

## HCV in LN

LN is a frequent complication of systemic lupus erythematosus (SLE), which manifests as immune complex glomerulonephritis (Yu et al., 2022). According to certain reports, the prevalence of HCV infection is significant among patients with SLE (Lo Presti et al., 2022). One study of 134 consecutive SLE patients and 200 consecutive volunteer blood donors, tested for HCV antibodies in the serum of all patients and controls, and revealed that 18 patients with SLE (13%) and 2 blood donors (1%) had detectable HCV antibodies in their serum (Ramos-Casals et al., 2000).

HCV is linked to the presence of autoimmune diseases, and patients with HCV infection often exhibit positive autoantibodies such as antinuclear antibody (ANA; 10%–30%), anticardiolipin antibody (ACL), antithyroid antibody, anti-smooth muscle antibody (ASMA), anti-liver and kidney microsome (anti-LKM) antibody, cryoglobulin or RF (Retamozo et al., 2022).

Anti-ribosomal (Rib)-P autoantibodies, along with anti-dsDNA and anticardiolipin antibodies, frequently coexist and serve as distinctive indicators of SLE (Choi et al., 2020). In one particular study, two samples were collected from male patients infected with HCV. One sample, named HCV-43, exhibited moderate binding to the C-terminus of Rib-P, while the other sample, called HCV-5, did not exhibit any binding to the C-terminus. However, ELISA demonstrated that HCV-5 exhibited responsiveness to U1-ribonucleoprotein (RNP), an autoantigen (Kessenbrock et al., 2007). HCV is believed to stimulate the generation of autoantibodies through activation of CD81 on B cells (Choi et al., 2020). The presence of anti-U1-RNP antibodies has been demonstrated prior to the onset of clinical symptoms in individuals with SLE (Boehncke and Brembilla, 2019). The research also utilized a structural model of the C-terminal Rib-P epitope and reported the first instance of developing anti-Rib-P autoantibodies within HCV infection. It was concluded that humoral autoimmunity to ribosomal P protein displays diversity in the context of autoimmunity and infection. While most of the anti-Rib-P positive samples bind to the C-terminal epitope and react to phenylalanine substitution, other linear and conformational epitopes may play a crucial role in the auto-immune response toward this protein complex. Notably, among 68 HCV-positive sera with anti-Rib-P2, two exhibited the same level of reactivity toward the epitope region within the N-terminal half of the P2 protein. Further investigations are deemed necessary to understand the potential role of HCV in initiating the self-response to ribosomal P protein and the onset of SLE (Kessenbrock et al., 2007).

LN can be caused by factors both within and outside the kidney. These factors are determined by various combinations of genetic variants that disrupt the body’s mechanisms for maintaining immune tolerance toward nuclear autoantigens. A breakdown in this tolerance is indicated by the presence of antinuclear antibodies in clinical tests (Lech and Anders, 2013). Approximately 1/5 to 2/5 of patients with HCV infection exhibit ANA seropositivity (Hsiao et al., 2022).

Furthermore, several clinical symptoms of systemic lupus resemble those of viral infections (Lech and Anders, 2013). A comprehensive study of 134 Spanish patients with SLE reported that the prevalence of HCV infection was similar to that of SLE (Tzang et al., 1999). In lupus, similar to viral particles during viral infection, endogenous nuclear particles activate interferon-α (IFN-α) signaling (Lech and Anders, 2013). Due to the suppression of cell-mediated immunity by some immunosuppressive drugs used to treat LN, these patients are prone to viral infections, which can lead to viral proliferation and liver damage (Mitwalli et al., 2012).

Patients with LN who are HCV-positive have a higher risk of CKD along with rapid deterioration of kidney function, increased rates of end-stage renal disease (ESRD) progression, and shorter survival times (Mitwalli et al., 2012). Between January 1995 and January 2008, a retrospective analysis was conducted at King Khalid University Hospital. The analysis involved a review of the medical records of 134 patients who were not undergoing dialysis and who had biopsy-confirmed World Health Organization (WHO) grade IV LN with chronic HCV infection. The study findings revealed a significant decrease in kidney function among patients with LN and HCV infection. Decreased kidney function ultimately leads to ESRD. Among patients without HCV infection, 95 and 80% survived without the need for dialysis after 5 and 10 years, respectively. Among those infected with HCV, 90 and 65% survived without dialysis at the end of 5- and 10-year periods, respectively (Mitwalli et al., 2012).

## HCV in RCC

RCC is a specific malignancy of the kidney, and according to data provided by the WHO, more than 140,000 RCC-related deaths occur each year, making this cancer the 13th leading cause of cancer-related deaths worldwide (Bahadoram et al., 2022).

The prevailing belief is that HCV primarily leads to liver cancer, but recent studies suggest that HCV may also increase the risk of RCC (Gordon et al., 2010). A study that conducted a search of the health system’s cancer registry for patients diagnosed with kidney cancer reported a diagnosis rate of 0.6% for HCV-positive patients (17 of 3,057) and 0.3% for HCV-negative patients (177 of 64,006) and noted that patients with HCV-positive RCC were considerably younger than those who were HCV-negative (Vallée and Lecarpentier, 2018).

Although various hypotheses have been proposed regarding the mechanism by which HCV causes RCC, this process is not completely understood. Some have speculated that a causal relationship may exist between HCV infection and NY-REN-54 RCC, possibly because NY-REN-54 impairs the autophagy response through the self-regulatory mechanism of the ubiquitin-protein ligase, which in turn drives cancer development (Wiwanitkit, 2011).

## The impact of HCV on outcomes of CKD patients and KT recipients

HCV-positive CKD patients have increased mortality, an increased risk of arrhythmias, acute myocardial infarction, acute coronary syndrome and transient ischemic attacks, and an increased incidence of cardiomyopathy and congestive heart failure. In addition, several studies have demonstrated that individuals with CKD are more likely to develop ESRD following HCV infection (Pol et al., 2019).

HCV infection also increases the likelihood of arterial disease in patients who have received a kidney transplant. Research has demonstrated that nondiabetic patients infected with HCV who undergo kidney transplantation have notably lower coronary flow reserve than patients not infected with HCV (Yelken et al., 2009). Moreover, HCV infection has been linked to decreased patient and graft survival rates after kidney transplantation, as HCV-positive kidney transplant recipients have higher risks of mortality and graft loss than HCV-negative recipients (El Helou et al., 2022). Additional information on the relationships between HCV and LT and KT is provided in Table 1.

**Table 1:** Treatment of diseases related to the liver and kidneys.

	Kidney disease	Treatment	Study subject	Outcomes	Reference
HCV (+) not transplanted	CKD	Grazoprevir + elbasvir; Glecaprevir + pibrentasvir	Clinical trial	99% SVR	Fabrizi et al. (2021)
	AKI	Sofosbuvir-based regimen	Clinical trial	95%SVR	Pozzato et al. (2020)
	GN	Antiviral treatment with a combination of standard or pegylated IFN with RBV; immunosuppressive therapy (including rituximab administration) followed by antiviral treatment	Clinical trial	SVR is achieved in 65%–90% of patients with genotypes 2 and 3, 30%–50% with genotype 1	Pipili et al. (2011)
HCV (+) KT	DN	SGLT2 inhibitors	Animal experiment	Improve hyperglycemia and slow the progression of diabetes-associated glomerulosclerosis and liver fibrosis	Tang et al. (2017)
	CKD	Sofosbuvir with ledipasvir, daclatasvir or simeprevir; Glecaprevir-pibrentasvir	Clinical trial	NA	Fabrizi et al. (2021)
	GN	α-IFN plus ribavirin therapy	Clinical trial	NA	Rostaing et al. (2012)
	DN	CyA, Insulin	Clinical trial	NA	Bloom et al. (2002)
HCV (+) LT	CKD	Immunosuppressive regimen accompanied by reduction or discontinuation of calcineurin inhibitors	Clinical trial	NA	Cabeza Rivera et al. (2023)
	AKI	Immunosuppressive therapy with MMF	Clinical trial	NA	Fabrizi et al. (2022)
	GN	Sofosbuvir and ribavirin	Clinical trial	Cryoglobulinemic symptoms improve	Fabrizi et al. (2019b)
	DN	mTORi	Clinical trial	Stabilize plasma levels of creatinine and renal function	Álamo et al. (2015)
HCV (+) LT and KT	CKD	ACE-inhibitors and ARBs	Clinical trial	Lower degree of liver stiffness	Marcuccilli and Chonchol (2016)
	AKI	Terlipressin	Clinical trial	Constrict blood vessels	Gupta et al. (2021)
HCV (+) HRS		Vasoconstrictor terlipressin, liver transplantation	Clinical trial	Terlipressin improves kidney function in patients with HRS, liver transplantation, which eliminates end-stage liver disease	Wong et al. (2021)

ACE, Angiotensin converting enzyme; AKI, Acute kidney injury; ARBs, Angiotensin receptor blocker; CKD, Chronic kidney disease; CyA, Cyclosporine A; DN, Diabetic nephropathy; GN, Glomerulonephritis; HCV, Hepatitis C virus; HRS, Hepatorenal syndrome; IFN, Interferon; KT, Kidney transplant; LT, Liver transplant; MMF, Mycophenolate mofetil; mTORi, mammalian target of rapamycin inhibitors; RBV, Ribavirin; SGLT2, Sodium-glucose cotransporter 2.

## Treatment and outcomes

The treatment objective in patients with HCV infection is to achieve a sustained virologic response (SVR), which is defined as the absence of detectable HCV RNA in serum 12 weeks after discontinuation of antiviral therapy (European Association for the Study of the Liver, 2017; Jadoul et al., 2018). Over the past decade, the introduction and gradual increase in the use of DAAs rather than protease and polymerase inhibitors has resulted in a SVR in a considerable number of individuals (Canonico et al., 2022). Patients with renal insufficiency can receive sofosbuvir-based therapy if their glomerular filtration rate (GFR) falls between <60 and >30 mL/min/1.73 m^2^. This treatment is administered in conjunction with peramivir/ritonavir/oseltamivir/dasabuvir or grazoprevir plus elbasvir. For patients with a GFR between <30 and >15 mL/min/1.73 m^2^, only the peramivir/ritonavir/oseltamivir/dasabuvir or grazoprevir plus elbasvir regimens are approved. In cases where alternative treatments are unavailable, sofosbuvir can be used with caution in patients with a GFR less than 30 mL/min. Finally, only a combination of grazoprevir plus elbasvir can be prescribed for patients who present with ESRD (GFR <15 mL/min/1.73 m^2^) or dialysis. The data required to make strong recommendations of specific treatments for patients with KT is insufficient, but when choosing a combination of drugs, potential interactions should be considered. The timing of HCV treatment is influenced by various factors such as the type of donor, expected wait times for different donors, the extent of liver fibrosis, and the patient’s willingness and readiness to receive an organ from an HCV-infected donor. Patients with compensatory cirrhosis or liver cirrhosis may need to wait longer than 24 weeks for transplants and can receive DAA treatment prior to transplantation to ensure SVR within 12 weeks post-transplantation. HCV-infected kidney transplant candidates with a designated living kidney donor may undergo HCV treatment either before or shortly after transplantation, based on the anticipated timing of the procedure (Martin et al., 2022). In the case of HCV-cryoglobulinaemia vasculitis (CryoVAS) with renal involvement, IFN-free DAA combination therapy may be recommended for patients with mesangial glomerulonephritis without severe morbidities. The duration of antiviral therapy can range from 3 to 6 months and depends on the DAA regimen and virological response predictors. In an in-depth analysis of extensive data collected from a clinical trial conducted over a significant timeframe, remarkable insights have been gained regarding the efficacy of DAA therapies in treating patients afflicted with HCV-CryoVas. The study meticulously reviewed the outcomes of a diverse patient cohort, all of whom were administered a range of DAA treatment regimens. The findings were both compelling and promising, revealing that an impressive over 95% of the patients experienced a noticeable alleviation of their symptoms, either in the form of complete remission or partial improvement. This high rate of symptom relief is a testament to the effectiveness of DAA treatments in managing the complex interplay of HCV and CryoVas (Cacoub et al., 2019). For patients who experience deterioration in renal function, a combination therapy of rituximab and concomitant DAA without IFN can be recommended. In cases of glomerulonephritis that progresses quickly due to digestive, cardiac, lung, and/or central nervous system issues, plasmapheresis can provide immediate benefits. It is necessary to combine plasmapheresis with immunosuppression to prevent postapheresis rebound of cryoglobulinaemia and to enhance the anti-B-lymphocyte activity of standard immunosuppressive drugs. However, based on open studies, rituximab used alone or in conjunction with methylprednisolone has demonstrated greater safety and efficacy (Roccatello et al., 2016; Cacoub et al., 2018). In the management of cryoglobulinaemic vasculitis, immunosuppression has been traditionally employed as a primary treatment option, particularly when significant kidney involvement is present, despite its potential to exacerbate the infection. For individuals experiencing cryoglobulinaemia or severe glomerular damage induced by HCV, the recommended course of action entails administration of rituximab, either with or without plasma exchange, until the acute phase of the disease subsides, followed by treatment with DAAs. In contrast, patients with mild or moderate HCV-induced glomerular disease should receive treatment consisting of DAAs. In cases where DAA treatment is ineffective or not well tolerated, the administration of immunosuppressive agents is warranted (Fabrizi et al., 2019a). In certain instances of cryoglobulinemic vasculitis (LoE 5C), a low dosage of rituximab (250 mg/mq weekly for 2 weeks) showed comparable effectiveness to a high dosage of rituximab (375 mg/mq/weekly for 4 weeks or 1 g 2 weeks apart). Research in the past primarily focused on treating mixed cryoglobulinemia-associated syndromes, non-Hodgkin’s lymphoma, and high doses of rituximab at 375 mg/m2 per week for 4 weeks. But some serious and life-threatening diseases should be preferred high-dose rituximab (Quartuccio et al., 2023). A recent comprehensive analysis of the patterns of relapse in patients with CryoVas has yielded significant findings. The study, which meticulously examined a range of clinical and demographic factors, has identified several key baseline risk factors that are independently associated with an increased likelihood of disease recurrence. Among these, male sex emerged as a significant predictor, suggesting that biological and hormonal differences may play a role in the disease’s progression and response to treatment. Additionally, the presence of skin ulcers was identified as another independent risk factor. This underscores the importance of skin manifestations in the overall assessment of CryoVas severity and the potential impact on patient outcomes. Furthermore, kidney involvement was also recognized as a critical risk factor for relapse. Renal complications in CryoVas are associated with a more severe disease course and poorer prognosis. The presence of renal involvement at baseline highlights the need for close monitoring and aggressive management of renal function in these patients (Fayed et al., 2022). Administering rituximab treatment during clinical relapse after the initial cycle proved to be both effective and safe for patients with severe but non-immediately life-threatening symptoms of cryoglobulin-related vasculitis (LoE 2B). Rituximab treatment cryoglobulinemic vasculitis has the vital significance, will now be rituximab and DAAs combination as the basic therapy for the treatment of patients with HCV related cryoglobulinemic vasculitis (Quartuccio et al., 2023).

Several studies have demonstrated that DAAs not only clear HCV but also reduce the risk of HCV-induced extrahepatic manifestations. HCV patients who achieve an SVR after DAA therapy have a lower risk of cryoglobulinaemic vasculitis and B-cell non-Hodgkin’s lymphoma than those who do not achieve an SVR (Cacoub et al., 2016). In addition, recent studies in the United States revealed that DAA treatment regimens can be employed safely during the perioperative phase of kidney transplantation to decrease the likelihood of HCV transmission. KT is safe in HCV-positive individuals and can increase the utilization of HCV-positive donor-transplanted organs (Doherty et al., 2022). DAA treatment to recipients of kidney transplants from HCV-infected donors who do not have HCV infection is considered safe and effective, both during and shortly after the transplantation process. This treatment has shown to effectively prevent complications related to HCV infection in recipients. Despite variations in the type of DAA regimens used and the timing of treatment initiation, studies have demonstrated that 4 to 8 weeks of DAA therapy leads to successful viral clearance (SVR12) and promotes optimal allograft function and survival, resulting in favorable patient outcomes one-year post-transplantation. Notably, a very short treatment duration (less than 8 days) has been linked with a higher risk of viral relapse; hence, a full 12-week course is typically recommended (Martin et al., 2022). HCV patients with extrahepatic manifestations who undergo kidney transplantation should be treated with DAAs as soon as possible, as these antiviral therapies can improve the survival rate of patients (Pol et al., 2019).

In addition to pharmacologic therapy, lifestyle modifications are essential for patients with kidney disease associated with HCV infection. 1. Smoking cessation. Smoking increases the risk of HCV infection and can promote chronic inflammation, which leads to renal failure and renal cell carcinoma. 2. Exercise. Studies have shown that high-intensity exercise reduces the risk of chronic kidney disease caused by HCV infection. 3. Healthy diet and adequate water intake. Diets should be high in fiber and protein and include moderate amounts of minerals such as iron, zinc and selenium, while sodium should be limited.

## Conclusion

HCV infection poses a significant global health challenge, as HCV causes both liver-related and non-liver-related health issues. This review discusses the relationship between HCV and different renal diseases. Excessive cryoglobulin production during HCV infection can lead to vasculitis and progression to AKI. HCV replicates in B lymphocytes and can trigger cryoglobulinaemia, which leads to MPGN. In one study, HCV positive individuals have a higher proteinuria and serum creatinine levels, suggesting that HCV infection may affect the progress of diabetic nephropathy. Some HCV-infected patients are seropositive for ANA, the presence of which indicates that the body’s immune tolerance to nuclear autoantigens has been disrupted, resulting in LN. NY-REN-54 disrupts autophagy through a self-regulatory mechanism involving ubiquitin protease, which promotes the development of RCC and may be related to HCV infection. In addition, HCV infection exerts adverse effects on the liver, kidney, and cardiovascular system in patients with CKD and KT. The connection between HCV and multiple kidney diseases underscores the importance of close collaboration between hepatologists and nephrologists.

## Author contributions

MZ: Writing – original draft. ZH: Writing – original draft, Writing – review & editing. YL: Writing – original draft. ZJ: Writing – original draft. SZ: Writing – original draft. SW: Writing – original draft. YT: Writing – original draft. JL: Writing – original draft. XL: Writing – review & editing. HC: Writing – review & editing.
